# Supplementary material for: Trends in summer presence of fin whales in the Western Mediterranean Sea Region: new insights from a long-term monitoring program
Source: PeerJ. 2020 Dec 14;8:e10544. doi: 10.7717/peerj.10544 (PMC7745674; doi:10.7717/peerj.10544)
Supplement: Supplemental Information 3 [file peerj-08-10544-s003.docx]

DATASET HABITAT REPRESENTATIVENESS

Representativeness of the sampled habitat has been tested. We used the ETOPO1 (bedrock)´ dataset from NOAA website (Amante & Eakins, 2009) to characterize bathymetry of the study area. We applied a 5x5 km^2^ grid to the study area and mean depth has been calculated for each cell. Cells crossed by tracklines have then been selected and flagged as Sampled cells.

Sampled depths were compared to overall area by looking at density plots of depths. Kruskal-Wallis test was then used to check differences in depth distributions between the area and the Sampled cells. Test has been run separately for each sub-region.

WP

For the Western Pelagos sub-region, Kruskal-Wallis test indicate no statistical difference between the overall sub-region and the sampled cells (Kruskal-Wallis chi-squared = 0.47404, df = 1, p-value = 0.4911). The little effort in the area indeed is taken into account when discussing results. It as to be underlined though that a good representation of preferred habitat of the species is given by the Sampled area, as both the slope area and the bathyal plan are well sampled by our effort.


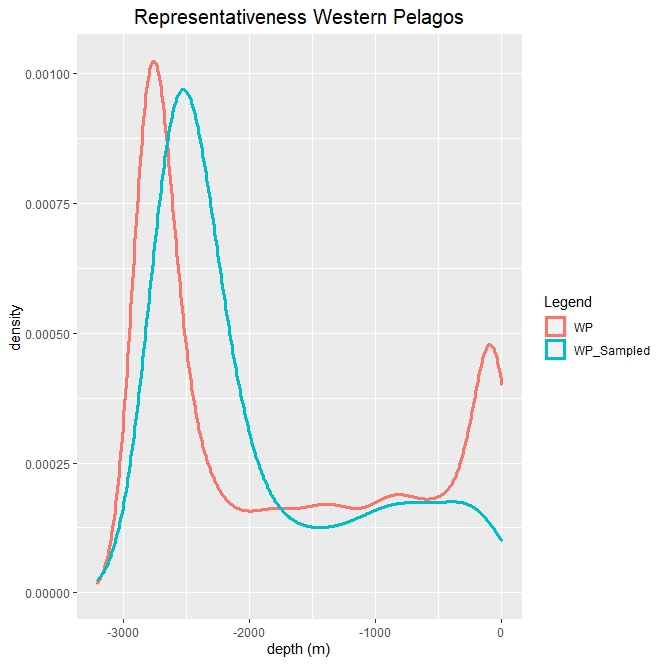


PEL

For the Pelagos Sanctuary, Kruskal-Wallis test indicate a statistical difference among the two considered datasets (chi-squared = 123.39, df = 1, p-value < 2.2e-16). Looking at the density plot of bathymetry, it emerges that main differences lie in the continental shelf region (depth > -500m), being this habitat little sampled by our effort.


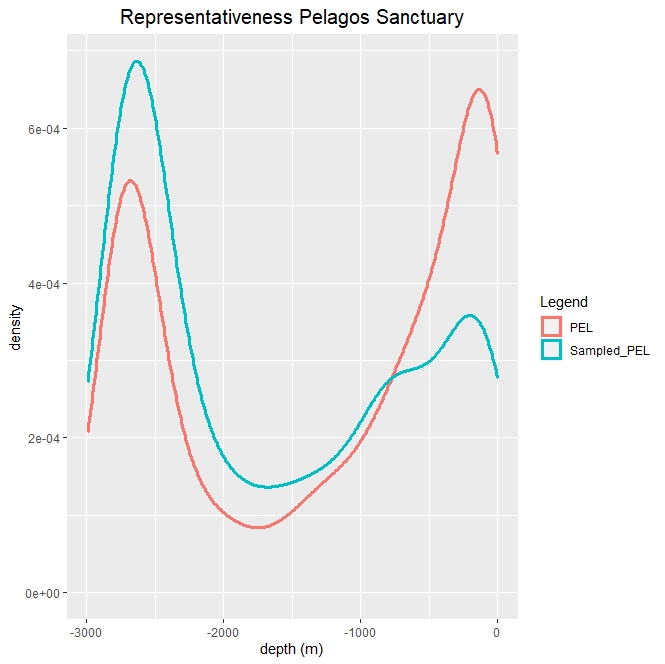


Kruskal-Wallis test run only considering depth < - 500m results in no statistical difference between the two datasets (chi-squared = 1.8032, df = 1, p-value = 0.1793). Considering that the continental shelf is not a preferred habitat for the species, we can consider our sampling effort as representative for this sub region

SEP

For the South Eastern Pelagos sub region, Kruskall-Wallis test indicate a difference between the sampled area and the overall considered subregion (chi-squared = 4.286, df = 1, p-value = 0.03843). Density plots indicate a lower coverage of the slope area and of depth greater than 3000m


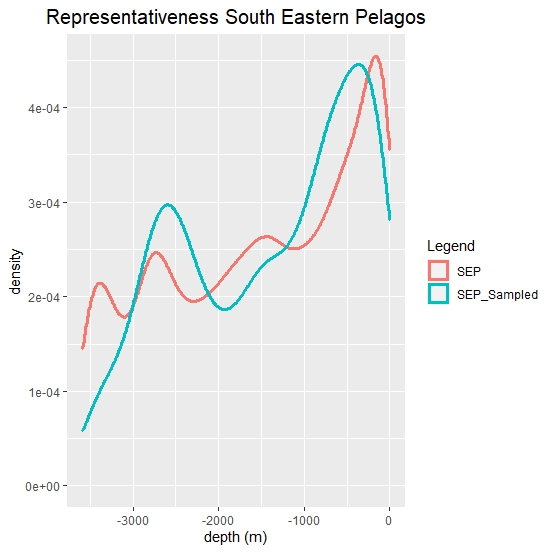


If we exclude the peak at depths > -3000m, the Kruskall-Wallis test confirm representativeness of the sampled dataset compared to the overall subregion (chi-squared = 2.3491, df = 1, p-value = 0.1254). We are aware of the importance of the deeper areas of the basin for the species and we consider that little effort in the area when discussing the results. Still, as mentioned in the manuscript, the effort in this subarea is one of the largest datasets for this region, as little information is usually available for this area.

Amante C, Eakins BW. 2009. *ETOPO1 1 Arc-Minute Global Relief Model: Procedures, Data Sources and Analysis. NOAA Technical Memorandum NESDIS NGDC-24.* DOI: 10.7289/V5C8276M.
