# Supplementary material for: Trends in summer presence of fin whales in the Western Mediterranean Sea Region: new insights from a long-term monitoring program
Source: PeerJ. 2020 Dec 14;8:e10544. doi: 10.7717/peerj.10544 (PMC7745674; doi:10.7717/peerj.10544)
Supplement: Supplemental Information 4 [file peerj-08-10544-s004.docx]

**Distance Sampling Analysis**

Distance sampling analysis has been performed using the package RDistance (version2.1.3) in R (version 3.6.1). The objective of the analysis was to compute the Effective Strip Width (ESW) separately for each different type of ferry used in the FLTMed Monitoring Network project.

Here we report the results of the Distance Sampling analysis. First, sightings data have been inspected for assessing the optimal truncation distance


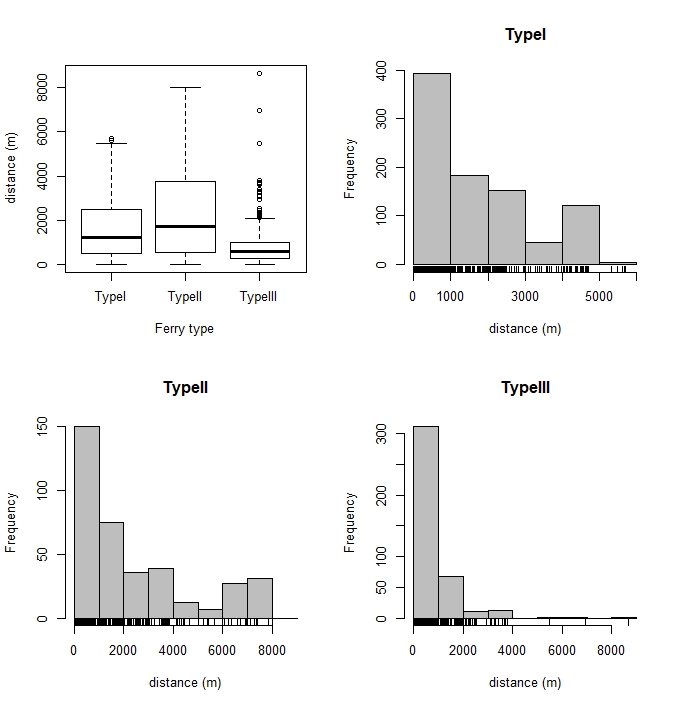


Figure 1_sup Box-Plot and histograms of frequency distribution of perpendicular distance for each different type of ferry

Type I Ferries do present few outliers, which have been excluded from the analysis and the cutoff distance has been set to 5000m.

Type II Ferries do not present outliers and the truncation distance has been set to 8000m

Type III Ferries do present some outliers, and the truncation distance has been set to 4000m.

For each type of ferry three different detection functions have been tested: Half normal, Uniform and Hazard rate, with zero or one adjustment. In order to choose the optimal detection function, the 6 obtained AIC have been compared and the best model has been chosen according to lowest AIC value.

Here we report all the results of the tested detection functions. In bold the final detection function and the relative ESW used in the analysis.

**TYPE I**

| Likelihood | Series | Expansion | AIC | ESW (m) | P(d) | Convergence |
| --- | --- | --- | --- | --- | --- | --- |
| Halfnorm | cosine | 0 | 15094.09 | 2067.585 | 0.61 | y |
| Halfnorm | cosine | 1 | 14992 | 2198.94 | 0.44 | y |
| Uniform | cosine | 0 | 15036.33 | 2423.244 | 0.48 | y |
| Uniform | cosine | 1 | 14978.83 | 1973.032 | 0.39 | y |
| **HazardRate** | **cosine** | **0** | **14969.24** | **1235.143** | 0.24 | y |
| HazardRate | cosine | 1 | 14988.54 | 2295.997 | 0.46 | y |

**TYPE II**

| Likelihood | Series | Expansion | AIC | ESW | P(d) | Convergence |
| --- | --- | --- | --- | --- | --- | --- |
| Halfnorm | cosine | 0 | 6637.074 | 4483.559 | 0.56 | y |
| Halfnorm | cosine | 1 | 6559.074 | 3011.397 | 0.38 | y |
| Uniform | cosine | 0 | 6594.29 | 3250.09 | 0.4 | n |
| Uniform | cosine | 1 | 6551.398 | 2656.601 | 0.33 | n |
| **HazardRate** | **cosine** | **0** | **6542.858** | **1415.164** | **0.18** | **y** |
| HazardRate | cosine | 1 | 6561.121 | 3129.123 | 0.4 | y |

**TYPE III**

| Likelihood | Series | Expansion | AIC | ESW | P(d) | Convergence |
| --- | --- | --- | --- | --- | --- | --- |
| Halfnorm | cosine | 0 | 6204.921 | 1359.761 | 0.34 | y |
| Halfnorm | cosine | 1 | 6166.439 | 1121.497 | 0.28 | y |
| Uniform | cosine | 0 | 6162.807 | 932.2162 | 0.233 | n |
| Uniform | cosine | 1 | 6160.415 | 943.8183 | 0.23 | n |
| **HazardRate** | **cosine** | **0** | **6160.746** | **1143.31** | **0.28** | **y** |
| HazardRate | cosine | 1 | 6175.018 | 1223.667 | 0.3 | y |

**Adding Group Size as a covariate**

For the selected functions, we then explore the role of Group size adding group as a covariate in the formula for the detection function.

| Type | Covariate | AIC | ESW | P(d) |
| --- | --- | --- | --- | --- |
| I | n | 14969.24 | 1235.143 | 0.24 |
| **I** | **y** | **14701.49** | **1858.554** | **0.37** |
| II | n | 6542.858 | 1415.164 | 0.18 |
| **II** | **y** | **6345.13** | **2656.58** | **0.33** |
| III | n | 6160.746 | 1143.31 | 0.28 |
| **III** | **y** | **5946.128** | **1112.617** | **0.28** |

**Considerations**

Looking at the histogram of frequency distributions of distances, an increase in frequency of individuals seen at higher distances is present. This partially violates the assumptions of Distance Sampling, where the probability of seeing the animals is expected to constantly decrease with perpendicular distance from the transect. In our case, we have an increase in probability of seeing animals at higher perpendicular distances, coinciding with the maximum linear distance at which animals have been seen. This anomaly is thought to be due to the fact that animals exactly on the heading of the ferry are more likely to be seen even at longer distances as the area in front of the command deck is the area with the best view, and the one covered partially by both observers.

We do not find the same anomaly in Type III ferries, where the detection probability falls drastically at 2000m, with some sightings occurring up to 4000m. This difference can also be due to the difference amount of sighting collected from this ferries, which are mainly operating in lower concentration areas for the species.

Adding group size as a covariates results in a lower AIC for the detection function for all Type of ferries so average ESW has been used in the analysis

The strip transect approach applied in the analysis though, as it applies a constant probability of detection p=1 within the ESW, is more robust to the effect of other parameters than conventional distance sampling, for which the computation of the probability detection function should take into account all these side effects (such as speed, influence of ferry in fin whales distribution, spatial displacement of transects)
